# Morin Prevents Non-Alcoholic Hepatic Steatosis in Obese Rats by Targeting the Peroxisome Proliferator-Activated Receptor Alpha (PPARα)

**DOI:** 10.3390/life14080945

**Published:** 2024-07-28

**Authors:** Laila Naif Al-Harbi

**Affiliations:** Department of Food Science and Nutrition, College of Food Science and Agriculture, King Saud University, Riyadh 11451, Saudi Arabia; lalharbi1@ksu.edu.sa

**Keywords:** diabetes, adipose tissue, fatty acid oxidation, fat mobilization, flavonoids

## Abstract

Background: Obesity has become a widespread issue globally. Morin, a flavonoid with traditional use in managing hyperglycemia and hyperlipidemia, has demonstrated antioxidant and anti-inflammatory properties in experimental studies. This research aims to explore the anti-obesity potential of morin in rats subjected to a high-fat diet (HFD) and investigate whether its effects are mediated through PPARα regulation. Methods: Young adult male Wistar albino rats were divided into four groups (*n* = 8/group): normal, morin (50 mg/kg/BWT, oral), HFD, and HFD + morin (50 mg/kg/BWT, oral). Treatments were administered daily for 17 consecutive weeks. Results: Morin mitigated the elevation in glucose levels and decreased fasting glucose and insulin levels, along with the HOMA-IR index, in HFD-fed rats. Furthermore, morin reduced calorie intake, final body weights, and the masses of subcutaneous, epididymal, peritoneal, and mesenteric fat in these rats. It also attenuated the rise in systolic blood pressure in HFD-fed rats and decreased serum levels of triglycerides, cholesterol, free fatty acids, LDL-c, and leptin, while increasing levels of HDL-c and adiponectin in both normal and HFD-fed rats. Moreover, morin restored normal liver structure and reduced fat vacuole accumulation in HFD-fed rats. Notably, it upregulated mRNA levels of PPARα in the livers and white adipose tissue of both normal and HFD-fed rats. Conclusions: These findings suggest the potential use of morin to enhance fatty acid oxidation in white adipose tissue and mitigate obesity, warranting further clinical investigation into its therapeutic applications.

## 1. Introduction

Obesity, marked by excessive adipose tissue and a high body mass index (BMI), is a critical global health issue [1]. It is linked with dyslipidemia, insulin resistance (IR), and impaired glucose metabolism, increasing the risk of type 2 diabetes mellitus (T2DM) and non-alcoholic fatty liver disease (NAFLD) [2]. Obesity also causes chronic low-grade inflammation with elevated inflammatory cytokines [3]. Non-metabolic risks include hypertension due to vascular function changes and an increased risk of cardiovascular diseases such as coronary artery disease and heart failure [4,5]. Comorbidities include musculoskeletal disorders like osteoarthritis and respiratory issues like obstructive sleep apnea [6]. Understanding these multifaceted risks is essential for effective prevention and treatment.

High-fat diets (HFDs) significantly contribute to obesity and NAFLD by creating an energy surplus, leading to increased adipose tissue and hepatic lipid accumulation [7,8,9]. These diets disrupt hormonal signaling, worsening appetite regulation and promoting overconsumption [10,11]. HFDs also alter the gut microbiota, exacerbating obesity and NAFLD [12]. Chronic inflammation from lipid overload further aggravates metabolic dysfunction and IR [13]. Addressing these mechanisms is crucial for developing targeted prevention and treatment strategies [14].

Regulating lipid metabolism in white adipose tissue (WAT) is key to managing obesity. Lipid synthesis in adipocytes involves hormonal, metabolic, and nutritional factors, balancing lipogenesis and lipolysis [15]. Existing anti-obesity drugs have been withdrawn due to adverse effects, underscoring the need for new approaches [16]. The peroxisome proliferator-activated receptor alpha (PPARα) is a key anti-obesity factor, regulating metabolism by reducing triglyceride synthesis and promoting fatty acid β-oxidation [17,18]. It activates anti-obesity genes like carnitine palmitoyltransferase I (CPT1), which is needed for fatty acid oxidation, and the uncoupling proteins (UCP1/2) [19]. PPARα has proven effects in reducing body weight, serum lipids, and fasting glucose levels, improving insulin sensitivity, and preventing hepatic steatosis and NAFLD in animal models [14,20].

Flavonoids, such as morin found in fruits, show promise in preventing metabolic disorders due to their antioxidant, hypoglycemic, hypolipidemic, and anti-inflammatory properties [21,22,23]. Morin, a yellow-colored flavonoid ubiquitous in various fruits, exhibits potent antioxidant activity, scavenging free radicals and mitigating oxidative-stress-induced damage [22]. Additionally, it demonstrates promising anti-inflammatory properties by inhibiting key inflammatory mediators and pathways in cell culture models [23]. Animal studies indicate morin’s potential in managing diabetes and hyperlipidemia [24], although human clinical trials are limited [25]. Previous research showed morin reduced hepatic lipogenesis and inflammation in HFD-fed rats by activating PPARα and downregulating inflammatory mediators [26]. In a previous study, morin was shown to alleviate hepatic lipogenesis and inflammation in rats fed an HFD for 8 months by downregulating lipogenic transcription factors and inflammatory mediators such as sterol regulatory element binding proteins (SREBPs) and NRLP3 inflammasome and activating PPARα [26]. However, the anti-obesity effect of morin remains elusive, and its potential to protect against obesity-induced NAFLD and reverse hepatic steatosis is yet to be elucidated.

This study aims to explore if morin supplementation can protect against obesity and hepatic steatosis in a chronic HFD rat model and examine its underlying mechanisms involving PPARα/CPT1 pathways.

## 2. Materials and Methods

### 2.1. Animals

Ten-week-old male Wistar rats weighing 150 ± 20 g were procured from the Experimental Animal Care Center at King Saud University (KSU) in Riyadh, Saudi Arabia. Ethical clearance for all experimental procedures involving these animals was obtained from the Research Ethics Committee at King Saud University, Riyadh, Saudi Arabia (Ethical Reference No: KSU-SE-23-30). The rats were housed under controlled environmental conditions, with humidity maintained at 60%, temperature set at 22 ± 5 °C, and a 12 h light/dark cycle. Before the experimental interventions, a one-week adaptation period was provided to allow the rats to acclimatize to their new environment. Following this period, they were randomly assigned to their respective experimental groups.

### 2.2. Induction of Obesity and NAFLD

Obesity induction in Wistar rats followed established methodologies documented in prior research [27]. This involved subjecting the rats to a 17-week regimen of chronic consumption of a high-fat diet (HFD). Both the standard diet (STD) (Catalog No. D12450K) and HFD (Catalog No. D12451) used in this study were sourced from Research Diets Inc., New Brunswick, NJ, USA. Detailed nutritional compositions of both diets are provided in Table 1. The HFD, specifically designed to induce obesity, is characterized by elevated levels of saturated fats and caloric density, essential factors in promoting adiposity in rodent models [27,28]. Before initiating the dietary intervention, all animals underwent an initial acclimatization period in a controlled environment to minimize potential stress-induced confounders. This meticulously controlled dietary manipulation serves as a foundational step in unraveling the underlying mechanisms implicated in diet-induced obesity and its associated metabolic dysregulations, thereby offering valuable insights into potential therapeutic interventions.

### 2.3. Preparation of Morin

Morin, procured as a finely ground powder from Sigma Aldrich (St. Louis, MO, USA) (Catalog No. M4008), was used in its pure form for the experiments. Prior to administration, morin was freshly dissolved in a 5% solution of carboxymethyl cellulose (5% CMC) to achieve the desired concentration of 50 mg/kg. This dosage selection was guided by a previous investigation conducted by Vanitha [29], which demonstrated the efficacy of this concentration in reducing fasting blood glucose levels in streptozotocin (STZ)-induced diabetic rats through the modulation of a critical enzyme involved in glucose synthesis.

### 2.4. Experimental Design

After completing the adaptation phase, rats were randomly assigned into groups of eight. The groups were delineated as follows: (1) the control group, where rats were fed the STD and orally administered the 5% CMC solution, which served as a vehicle; (2) the morin treatment group, consisting of rats fed the STD and orally administered a morin solution (dissolved in 5% CMC) at a dosage of 50 mg/kg/BWT; (3) the high-fat diet (HFD) group, in which rats were fed a high-fat diet and orally administered 5% CMC; and (4) the HFD + morin treatment group, where rats were fed a high-fat diet and orally administered the morin solution at the same dose (50 mg/kg/BWT). Throughout the 17-week treatment period, rats received CMC (as a vehicle) or morin (dissolved in CMC) via gavage using a stainless-steel feeding cannula, with dosing occurring twice weekly [29]. Body weights and energy intake were monitored every two weeks, with final measurements recorded after week 17. Rats were individually housed in separate cages for the duration of the study to prevent any confounding social interactions. Changes in body weight were assessed biweekly, and calorie intake (kcal/g/day) was calculated using the following formula: daily food consumption (g) multiplied by the total energy content of food (kcal/kg), divided by 1000 [30].

### 2.5. Measurement of Systolic Blood Pressure

During the final phase of the experimental period, rats were subjected to a gradual familiarization process over seven days, wherein they were introduced daily to the handling procedures and protocols essential for the accurate measurement of systolic blood pressure (SBP). On the seventh day, SBP assessment was conducted using a non-invasive tail-cuff method, employing specialized equipment (Powerlab, ADInstruments, Bella Vista, Australia), following established protocols outlined in prior publications [30]. To ensure optimal conditions for measurement, rats were first placed in an environment maintained at a constant temperature of 37 °C for 10 min, aimed at enhancing the detectability of the tail arterial pulses. Following the stabilization of pulse readings, SBP was determined as the average of five consecutive trials, each executed with meticulous attention to detail and adherence to standardized procedures.

### 2.6. Oral Glucose Tolerance Test (OGTT)

On the final day of the 17-week study period, rats were subjected to an overnight fasting regimen before undergoing an oral glucose tolerance test (OGTT), following the protocol established by Ghezzi [31]. Each rat received a glucose solution of 2 g/kg via gavage administration. Blood samples were obtained from the tails of gently restrained rats, where a minor incision was made at the tip following local anesthesia application (Marcaine (0.5%; 5 mg/mL). These samples were collected at 0, 15, 30, 60, and 120 min intervals in EDTA-treated Eppendorf tubes and subsequently centrifuged at 1200× *g* for 10 min to separate plasma. The measurement of blood glucose and insulin levels was performed using assay kits (Cat No. 10009582, Cayman Chemicals, Ann Arbor, CA, USA, and Cat. No. 589501, Ann Arbor, TX, USA). IR in rats was assessed by calculating the homeostasis model assessment (HOMA) according to fasting glucose and insulin levels using the following formula: glucose (mg/dL) × insulin (ng/mL)/405 [27]. All analyses were conducted in duplicate for each sample, with a total of eight samples analyzed per experimental group following the manufacturer’s instructions for each kit.

### 2.7. Collection of Tissues and Blood Sampling

Upon completion of the experimental protocol, rats underwent an overnight fasting period before being anesthetized using a mixture of ketamine and xylazine (80/10 mg/kg). Blood samples were then obtained via cardiac puncture and collected in plain tubes. Following a 30 min clotting period at room temperature, the samples were centrifuged at 1200× *g* for 10 min to separate serum, which was subsequently stored at −20 °C for further analysis. Ethical procedures were followed for euthanasia, involving neck dislocation. Liver and various fat pad types, including subcutaneous (inguinal), epididymal, peritoneal, and mesenteric, were harvested from each rat, weighed, and promptly stored at −80 °C until further experimentation.

### 2.8. Biochemical Measurements in the Serum

The serum of each rat was analyzed for various parameters using specific enzyme-linked immunosorbent assay (ELISA) kits designed for rats. Tumor necrosis factor-alpha (TNF-α), interleukin-6 (IL-6), total adiponectin, leptin, and albumin levels were measured using respective ELISA kits (Cat. No. BMS622, ThermoFisher, Karlsruhe, Germany; Cat. No. R6000B, R&D Systems, Minneapolis, MN, USA; Cat. No. ab239421, Abcam, Cambridge, UK; Cat. No. ab100773, Abcam, Cambridge, UK; Cat. No. 80662, Crystal Chemicals, CA, USA). Additionally, commercial assay kits were utilized to assess serum TGs, cholesterol (CHOL), low-density lipoprotein cholesterol (LDL-c), and free fatty acid (FFA) levels (Cat. No. ECCH-100, BioAssay Systems, Hayward, CA, USA; Cat. No. 10009582, Cayman Chemicals, Ann Arbor, CA, USA; Cat. No. 79960, Crystal Chemicals, USA; Cat. No. MBS014345, MyBioSource, San Diego, CA, USA). All measurements were conducted per the manufacturer’s instructions, with duplicate readings obtained for each sample using the SpectroSTAR Nano plate reader (Allmendgrün, Germany). Each analysis included samples from eight rats per experimental group.

### 2.9. Biochemical Analysis in the Liver Homogenates

Biochemical analysis of liver samples was performed to assess various parameters. Frozen liver tissue (approximately 100 mg) was homogenized in 0.5 mL ice-cold phosphate-buffered saline (pH = 7.4) at a ratio of 10:1 (buffer–tissue). The homogenates were then subjected to centrifugation at 1200× *g* for 10 min at 4 °C. The resulting supernatants were carefully collected and stored at −80 °C until further use. Subsequently, reduced glutathione (GSH), superoxide dismutase (SOD), and malondialdehyde (MDA) levels were determined using commercially available assay kits (Cat. No. MBS265966, Cat. No. MBS036924, and Cat. No. MBS268427, respectively; MyBioSource, CA, USA) following the manufacturers’ instructions. Spectrophotometric readings for all assays were performed using the SpectroSTAR Nano plate reader (Allmendgrün, Germany). Each measurement was conducted in duplicate for each sample, with a total of eight samples analyzed per experimental group.

### 2.10. Real-Time RT-qPCR

qPCR was conducted to measure the mRNA levels of PPARα and CPT1 in the liver and WAT (epididymal fat). The primer sequences for PPARα (NM_019142) and GAPDH (NM_017008.3) (a reference gene) were adopted from the study by Yang et al. [32] as follows: PPARα (forward: TGCGGACTACCAGTACTTAGGG; reverse: GCTGGAGAGAGGGTGTCTGT); GAPDH (forward: GAGATCAACGTGTTCCAGTGC; reverse: CTTCCACCACGTAGGGATTC). The primers for CPT1 (NM_013200.1) were based on research by Al Jadani et al. [33] (forward: TCCGAGGCAGGAGCCCCATC; reverse: TCTCGGTCCAGTTTGCGGCG). The total RNA was isolated from samples using a commercial isolation kit (Cat. No. 74004; Qiagen, Hilden, Germany), and the first-strand cDNA was synthesized using the supplied commercial kit (Cat. No. K1621, Thermo Fisher kit). The integrity and concentration of isolated RNA were confirmed by measuring the absorbance at 260/280 nm using a nanodrop spectrophotometer. Then, the qPCR was conducted in a Bio-Rad qPCR amplification machine with the help of the Ssofast Evergreen Supermix kit (Cat. No. 172-5200, from BioRad, USA). Amplification was carried out in 20 μL of the reaction mixture/well, containing the following ingredients: 10 μL of Ssofas Evergreen master mix reagent, 0.2 µL of the forward primer (final concentration: 500 nM), 0.2 µL of the reverse primer (final concentration: 500 nM), 2 μL of template cDNA (final concentration: 50 ng), and 7.6 µL of nuclease-free water. The following conditions were used for the amplification: heating (1 cycle/98 °C/30 s), denaturation (40 cycles/98 °C/5 s), annealing (40 cycles/60 °C/5 s), and melting (1 cycle/95 °C/5 s/step). The relative mRNA expression of PPARα was presented after the normalization of GAPDH. The ratio of expression was performed using the available software and following Livak’s double delta Ct method [34]. All procedures were performed as instructed by each kit manufacturer.

### 2.11. Histology Study

In this study, we employed a meticulous protocol for staining liver tissue sections with hematoxylin and eosin (H&E) to visualize cellular structures and evaluate histological changes. Following tissue fixation, liver sections were dehydrated through a series of graded alcohols and embedded in paraffin wax. Subsequently, tissue sections were cut into thin slices (4–5 μm) using a microtome and mounted onto glass slides. The sections were then deparaffinized in xylene and rehydrated through descending grades of alcohol. Next, the sections were immersed in hematoxylin solution for nuclear staining, followed by differentiation in acid alcohol and bluing in running tap water. Counterstaining was performed using an eosin solution to highlight cytoplasmic components. Finally, the sections were dehydrated, cleared in xylene, and cover-slipped using a mounting medium. All staining procedures were conducted meticulously to ensure optimal visualization of liver tissue architecture and cellular morphology. All tissue was examined and photographed under a light microscope (model No. ECLIPS Ni/Ci). This protocol was adapted from standard histological techniques [35].

### 2.12. Statistical Analysis

Data analysis was performed using GraphPad Prism analysis software (Version 8, San Diego, CA, USA). The normality of the data was assessed using the Kolmogorov–Smirnov test. Statistical analysis was conducted using a two-way ANOVA followed by Tukey’s test for post hoc analysis to adjust for multiple comparisons (*p* < 0.05). Results were presented as means ± standard deviation (SD).

## 3. Results

### 3.1. Changes in Food and Calorie Intake and Body Weight

**Figure 1:**
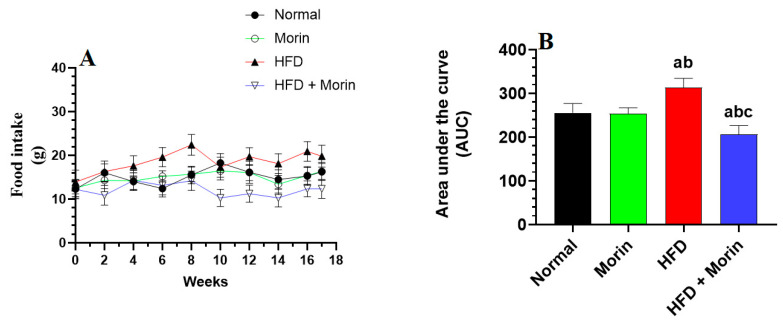

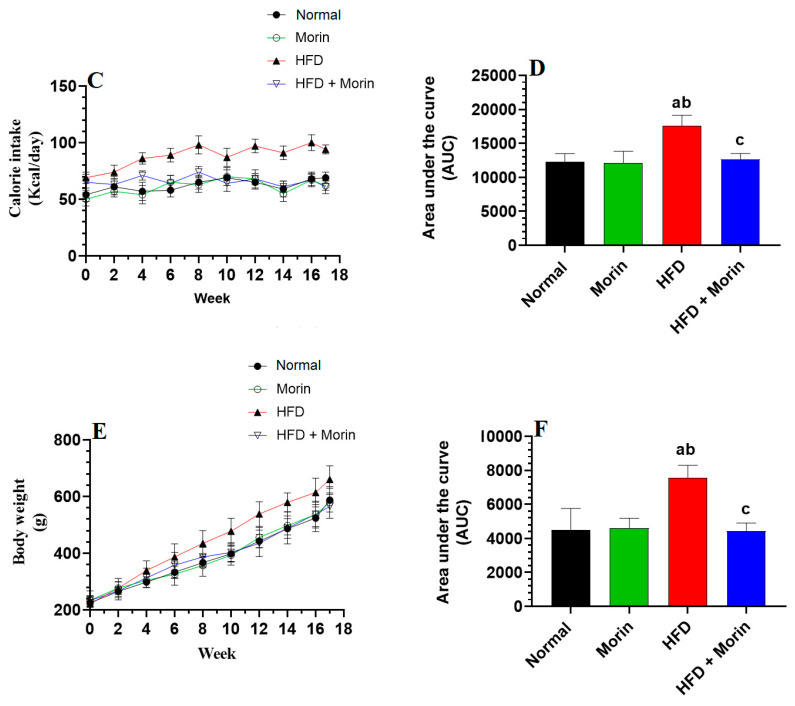
Food intake (**A**), calorie intake (**C**), changes in body weights (**E**), and their respective areas under the curve (**B**,**D**,**F**, respectively) were assessed in all groups of rats throughout the entire 17-week study period. The data are expressed as means ± standard deviation (*n* = 8/group). Significance levels are denoted as follows: a: compared to normal; b: compared to morin-treated normal rats (50 mg/kg); c: compared to HFD-fed rats.

HFD-fed rats showed a progressive increase in their food and energy intake, as well as in their body weights, starting from week 2 to week 17 of the study. There were no significant changes in food intake, energy intake, or body weights between the control rats and the control group, which was fed morin (Figure 1A–C). Opposing to HFD-fed rats, the HFD + morin-treated animals showed a significant reduction in their food and energy intake, as well as in their weights, all during the 17 weeks of the study and starting from week 4. Even, though the gain in body weight and energy intake were not significant, these rats showed a slight but significantly lower food intake as compared to control or control + morin-treated rats (Figure 1A–C).

### 3.2. Changes in Fat Pads

**Figure 2:**
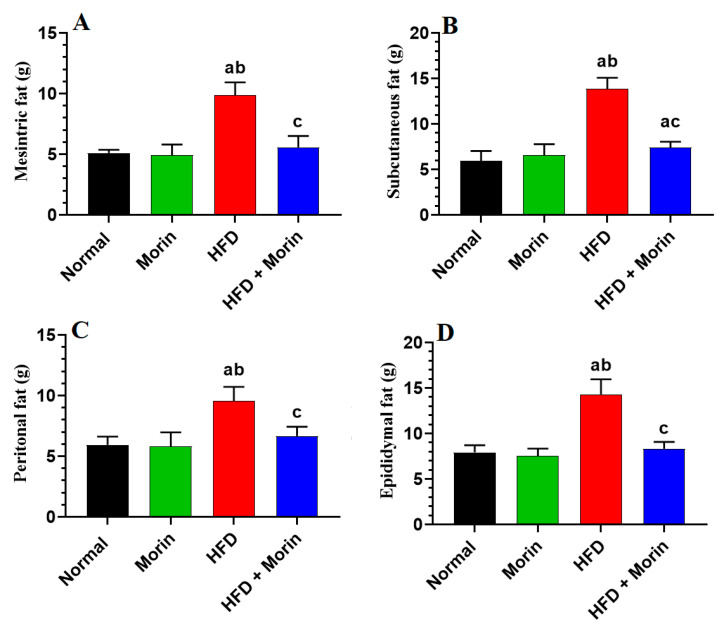
Mesenteric fat (**A**), subcutaneous fat (**B**), peritoneal fat (**C**), and epididymal fats (**D**).The weights of various fat pads were measured across all rat groups. Data are expressed as means ± SD (*n* = 8/group). Statistical comparisons were made as follows: a: versus normal; b: versus morin-treated normal rats (50 mg/kg); c: versus HFD-fed rats.

The weights of the mesenteric, subcutaneous, peritoneal, and epididymal fats showed no significant differences between the normal and morin-treated rats at the conclusion of the study (*p* < 0.05). In contrast, these fat deposits were markedly increased in HFD-fed rats (*p* < 0.001) (Figure 2A–D). However, in HFD + morin-treated rats, the weights of these fat depots were significantly reduced compared to the HFD-fed rats (*p* < 0.001). Notably, only the levels of subcutaneous fat remained significantly higher in the HFD + morin group compared to the normal and morin-treated groups (*p* < 0.01) (Figure 2A–D).

### 3.3. Changes in Parameters of Glucose after OGTT

**Figure 3:**
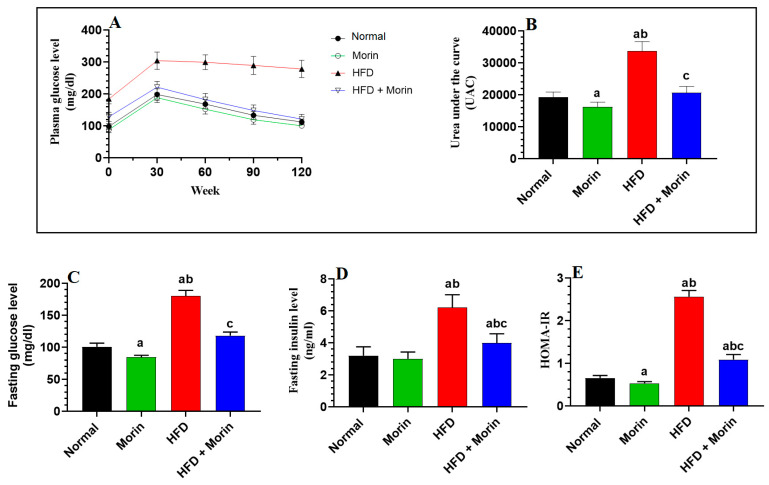
Plasma Glucose level (**A**), Urea under the curve (**B**), Fasting glucose level (**C**), Fasting insulin level (**D**), HOMA-IR (**E**). Blood glucose levels during the oral glucose tolerance test (OGTT) are depicted in panel **A**, with the corresponding area under the curve (AUC/B). Additionally, fasting levels of glucose, insulin, and HOMA-IR are presented for all groups of rats. The data are expressed as means ± SD (*n* = 8/group). Comparisons are indicated as follows: a: vs. normal; b: vs. morin-treated (50 mg/kg) normal rats; c: vs. HFD-fed rats.

Glucose levels exhibited a significant increase 30 min following glucose administration across all rat groups (Figure 3A). However, in HFD-fed rats, glucose levels measured at 30, 60, 90, and 120 min did not exhibit significant variation among each other, whereas they gradually decreased and were statistically significant (*p* < 0.05) in all other groups (Figure 3A). Analysis of the area under the curve (AUC) for glucose levels during the oral glucose tolerance test (OGTT) revealed no significant variation between normal and morin-treated rats (*p* > 0.05), while it was notably higher in HFD-fed rats (*p* < 0.001) (Figure 3A). Conversely, the AUC measured in HFD + morin-fed rats was significantly lower than in HFD-fed rats (*p* < 0.001), showed no significant change compared to normal rats (*p* > 0.05), and was significantly higher than in morin-treated rats (*p* < 0.001) (Figure 3B).

### 3.4. Changes in Fasting Glucose, Insulin, and HOMA-IR

Fasting levels of glucose and insulin, alongside HOMA-IR, demonstrated a notable increase in HFD-fed rats compared to normal rats (*p* < 0.0001) (Figure 3C–E). Conversely, in both morin-treated and HFD + morin-treated rats, the levels of these markers exhibited a significant reduction compared to normal and HFD-fed rats, respectively (*p* < 0.001) (Figure 3C–E). However, fasting glucose, fasting insulin, and HOMA-IR levels remained significantly higher in HFD + morin-treated rats than in normal rats (*p* < 0.01) (Figure 3C–E).

### 3.5. Changes in SPB and Serum Markers

SBP did not show significant differences between the normal and morin-treated rats (*p* > 0.01) but was notably elevated in HFD-fed rats (*p* < 0.001), as indicated in Table 1. Conversely, serum levels of TGs, CHOL, LDL-c, and FFAs were significantly reduced, while HDL-c levels were significantly depleted in morin-treated rats compared to normal rats (*p* < 0.01). SBP values were lower in HFD + morin-treated rats compared to normal rats (*p* < 0.01), as outlined in Table 2. Moreover, serum levels of TGs, CHOL, LDL-c, FFAs, leptin, TNF-α, and IL-6 were markedly increased, while HDL-c and adiponectin levels were significantly decreased in HFD-fed rats compared to normal rats (*p* < 0.0001), as presented in Table 2. Conversely, serum levels of TGs, CHOL, LDL-c, FFAs, leptin, TNF-α, and IL-6 were substantially reduced, and HDL-c and adiponectin levels were significantly increased in morin and HFD + morin-treated rats compared to HFD-fed rats (*p* < 0.001), as indicated in Table 2.

### 3.6. Changes in Hepatic Oxidant/Antioxidant Markers

**Figure 4:**
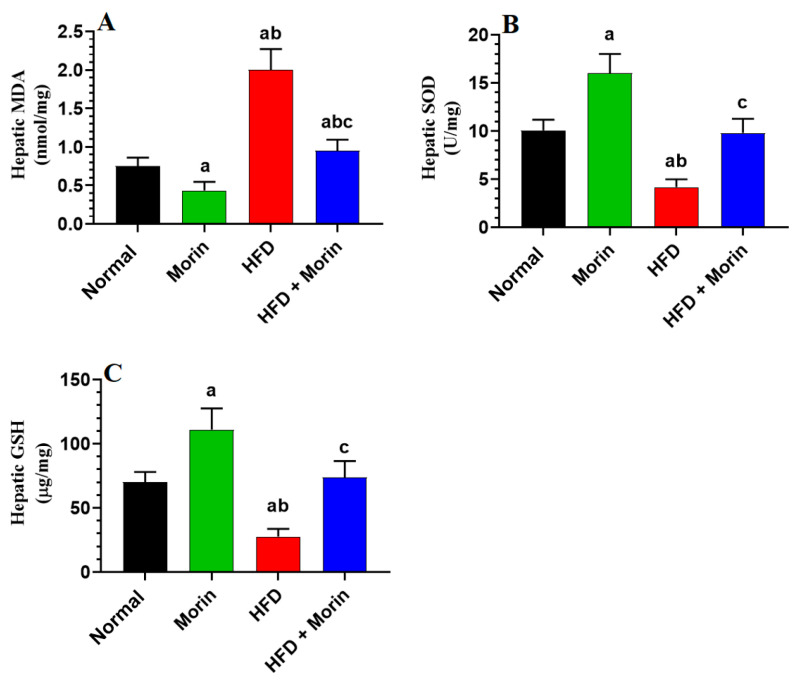
The levels of malondialdehyde (MDA) (**A**), total superoxide dismutase (SOD) (**B**), and total glutathione (GSH) (**C**) in the livers of all rat groups were assessed. Data are expressed as means ± standard deviation (SD) with eight rats per group. Statistical comparisons were made as follows: a, compared to normal rats; b, compared to morin-treated normal rats (50 mg/kg); c, compared to HFD-fed rats.

Hepatic concentrations of MDA exhibited a significant increase, whereas levels of SOD and GSH were notably decreased in the livers of HFD-fed rats compared to normal rats (*p* < 0.0001), as depicted in Figure 4A–C. Conversely, MDA levels were significantly reduced, while levels of GSH and SOD were significantly increased in the livers of both normal and HFD-fed rats treated with morin compared to their respective controls (*p* < 0.001), as illustrated in Figure 4A–C. Although MDA levels remained slightly elevated and statistically significant, the levels of SOD and GSH did not exhibit significant variation between normal and normal + HFD-fed rats (*p* < 0.05), as shown in Figure 4A–C.

### 3.7. Changes in the Expression of FA Oxidation-Related Genes

**Figure 5:**
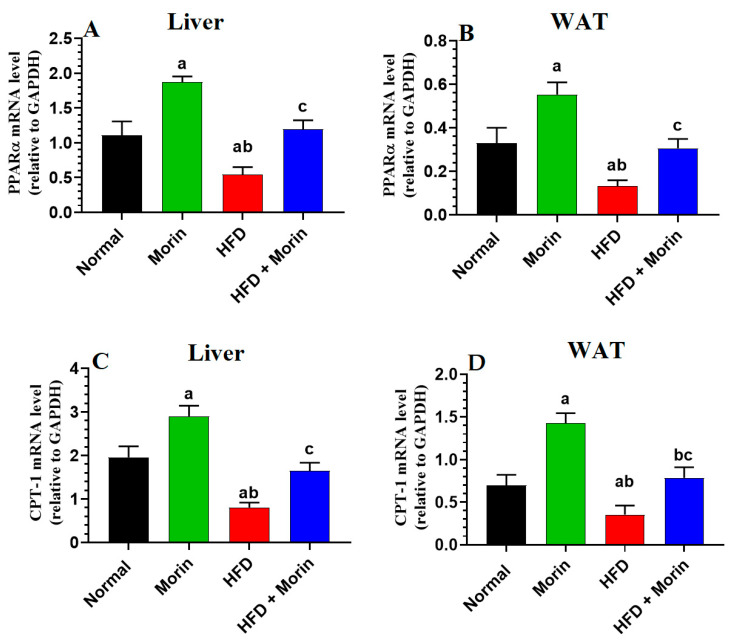
mRNA levels of PPARα and CPT-1 in the liver (**A**,**C**) and white adipose tissues (WAT) (**B**,**D**) of all groups of rats. Data are presented as means ± SD (*n* = 8/group). ^a^: vs. normal; ^b^: vs. morin-treated normal rats (50 mg/kg); ^c^: vs. HFD-fed rats).

The expression levels of PPARα and CPT-1 mRNA were notably diminished in the livers and white adipose tissue (WAT) of rats fed a high-fat diet (HFD) compared to those of normal rats (*p* < 0.05) (Figure 5A–D). Conversely, the mRNA levels of both genes exhibited a significant increase in the livers of rats treated with morin, as well as those treated with both morin and HFD, in comparison to normal rats or HFD-fed rats, respectively (*p* < 0.001) (Figure 5A–D). Importantly, there were no significant differences in PPARα and CPT-1 expression between the groups of rats treated with a normal diet, morin alone, or morin in combination with HFD (*p* > 0.05) (Figure 5A–D).

### 3.8. Histological Findings

**Figure 6:**
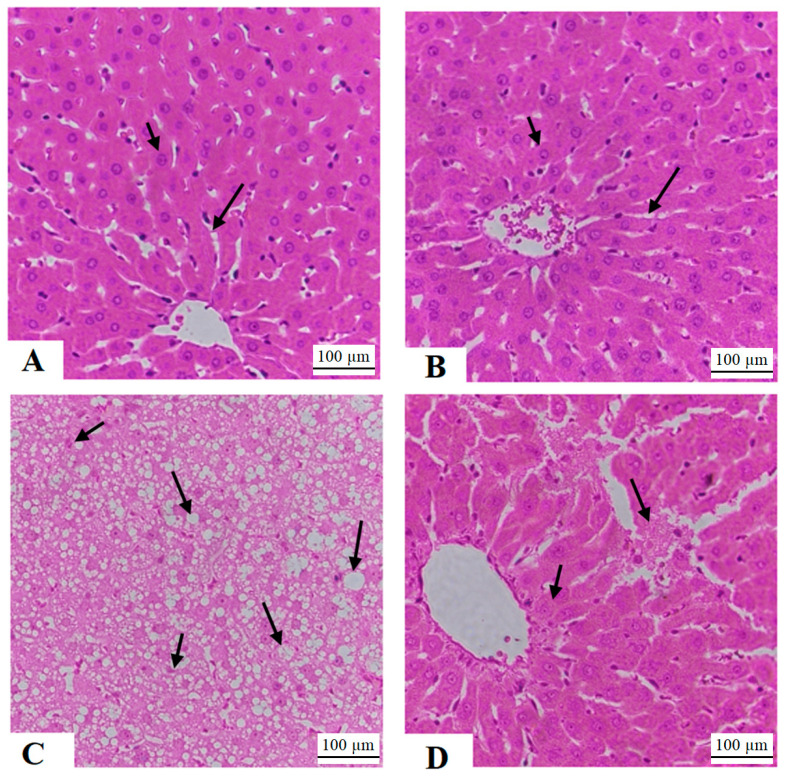
Liver histology was examined across all rat groups. (**A**,**B**) represent samples from normal rats and those treated with morin, respectively, depicting typical features such as hepatocytes (short arrow), the central vein (CV), and sinusoids (long arrow). (**C**), derived from an HFD model rat, illustrates pronounced and severe fatty vacuoles within hepatocyte cytoplasm (long arrow). In contrast, (**D**), from an HFD + morin-treated rat, displays considerable improvement in liver structure. Most hepatocytes appear normal (short arrow), with few fat vacuoles present. However, some regions exhibit signs of cell necrosis (long arrow).

The livers of rats treated with either a normal diet or morin displayed typical characteristics, depicting hepatocytes arranged around the central vein with intact sinusoids (Figure 6A,B). Conversely, livers from rats fed a high-fat diet (HFD) exhibited an abundance of cytoplasmic fat vacuoles of various sizes (Figure 6C). In contrast, the livers of rats treated with both HFD and morin showed mostly normal features, albeit with occasional necrotic hepatocytes observed in certain areas (Figure 6D).

## 4. Discussion

Over the past decade, the incidence of obesity has significantly increased, primarily due to changes in dietary habits. This rise has underscored the limited efficacy of existing anti-obesity medications and highlighted the urgent need for novel therapeutic approaches. Our study presents new evidence supporting the anti-obesity potential of morin, a flavonoid, in a rodent model. Morin demonstrated significant effects independent of dietary intake or energy consumption, including reductions in body weight and fat deposits, improved lipid and glucose levels, and decreased inflammatory markers. These benefits are attributed to the activation of free fatty acid (FFA) oxidation through PPARα stimulation in both liver and adipose tissues.

Experimental rodent models of obesity, induced by high-fat diets (HFDs) with 40% to 60% fat, closely mimic human obesity and related metabolic disorders [35]. This model results in rapid weight gain, increased food consumption, and metabolic changes akin to type 2 diabetes, such as hyperglycemia, insulin resistance (IR), and dyslipidemia. Various factors, including age, sex, and strain, affect obesity susceptibility, with younger and male rats, particularly Wistar rats, showing heightened vulnerability [35,36,37]. Our study employed young adult male Wistar rats and validated the HFD model by documenting increases in body weight, adipose tissue mass, and adverse metabolic markers, consistent with previous research [30].

Morin’s effects were striking: it reduced body weight, food intake, fasting glucose levels, and normalized lipid profiles. Morin also alleviated hepatic steatosis by reducing fat accumulation in the liver and preserving liver cell integrity. This aligns with prior studies demonstrating morin’s hypoglycemic and hypolipidemic effects in both diabetic and non-diabetic models [29,38,39,40]. Notably, the reduction in body weight observed may be partly due to decreased food intake, suggesting that morin might reduce appetite. However, the lack of effect on food intake in control rats points to additional mechanisms, such as enhanced lipolysis and fat oxidation in adipose tissue [41,42].

The precise mechanisms by which morin exerts its effects remain unclear. Prior research suggests that morin’s hypoglycemic effects stem from inhibiting gluconeogenesis enzymes and activating glycolysis enzymes [29]. Morin also antagonizes liver X receptors, crucial for cholesterol synthesis and insulin sensitivity [40], and inhibits protein tyrosine phosphatases that impair insulin signaling [38]. Further studies are necessary to fully elucidate how morin influences lipid and glucose metabolism.

Adipogenesis, driven by hormonal signals, regulates fat cell differentiation and adipose tissue function, affecting metabolism and satiety [43]. Obesity is associated with inflammation and elevated cytokines such as TNF-α and IL-6, which can lead to tissue damage and contribute to conditions like NAFLD [44,45]. Conversely, adiponectin enhances insulin sensitivity and protects against NAFLD by reducing hepatic lipogenesis [46]. Our study observed increased TNF-α, IL-6, and leptin levels, along with decreased adiponectin, in HFD-fed rats, correlating with increased body weight and metabolic dysfunction. Morin treatment reduced these inflammatory markers and improved adiponectin levels, indicating its potential to modulate inflammation and enhance metabolic health.

Oxidative stress, characterized by excessive ROS production, contributes to the progression of NAFLD to NASH [47]. Antioxidants and anti-inflammatory agents are crucial in preventing this progression [48]. Morin reduced lipid peroxidation and increased antioxidant levels (GSH and SOD) in HFD-fed livers, reflecting its potent antioxidant properties [49]. Morin also mitigated ROS-induced apoptosis and preserved mitochondrial integrity [50], consistent with its protective effects against oxidative damage [51].

Hypertension, a common obesity-related complication, is associated with metabolic syndrome. Our study found that a high-fat diet increased systolic blood pressure (SBP), but morin treatment reduced SBP in HFD-fed rats, suggesting its potential as a hypotensive agent.

PPARα, a key regulator in metabolically active tissues like the liver and muscles, plays a critical role in lipid metabolism. PPARα activation promotes FA oxidation and reduces TG synthesis, which helps alleviate obesity [52]. Our study found decreased PPARα and CPT1 levels in HFD-fed rats, but morin enhanced their expression in both normal and HFD-fed rats. This suggests that morin may counteract obesity by promoting FA oxidation and reducing TG availability, offering new insights into its anti-obesity effects [53].

While these findings are significant, they are observational and require further validation. Future studies should explore morin’s effects in female rats and PPARα-deficient models. Additionally, investigating other lipid regulatory pathways, such as SREBP1/2 and PPARγ, could provide a more comprehensive understanding of morin’s therapeutic potential. Research into morin’s toxicity has shown it to be generally safe, but further clinical trials are needed to confirm its safety and efficacy [54,55,56,57].

## 5. Conclusions

This study shows that morin is an excellent adjuvant drug option that can be used to treat obesity and its associated complications. The anti-obesity effects of morin seem to be related to its hypolipidemic effect mediated by its action as a PAPRα agonist, which stimulates FA oxidation. These data encourage further trials at preclinical and clinical levels with the hope of achieving positive results.

## Figures and Tables

**Table 1:** Composition of normal and HFD.

	Normal Diet (D12450K)		HFD Diet (D12450K)	
	gm%	kcal%	gm%	kcal%
Protein	19.2	20	24	20
Carbohydrates	67.3	70	41	35
Fat	4.3	10	24	45
Toral energy (kcal/g)	3.85		4.73	
Ingredients				
Casein, 30 mesh	200	800	200	800
L-Cysteine	3	12	3	12
Corn starch	550	2200	72.8	291
Maltodextrin 10	150	600	100	400
Sucrose	0	0	172.8	691
Cellulose, BW200	50	0	50	0
Soybean Oil	25	225	25	225
Lard *	20	180	177.5	1598
Mineral Mix S10026	10	0	10	0
DiCalcium Phosphate	13	0	13	0
Calcium Carbonate	5.5	0	5.5	0
Potassium Citrate, 1 H_2_O	16.5	0	16.5	0
Vitamin Mix V10001	10	40	10	40
Choline Bitartrate	2	0	2	0
FD&C Red Dye #40	0.025	0	0.025	0
FD&C Blue Dye #1	0.025	0	0.025	0
Total	1055.05	4057	858.5	4057

* Typical analysis of cholesterol in lard = 0.95 mg/g. Cholesterol (mg)/4057 kcal = 168.6 cholesterol (mg)/kg = 196.5.

**Table 2:** Lipid marker profiles in all groups of rats.

Parameter	Normal	Morin	HFD	HFD + Morin
SBP (mmHg)	102.4 ± 11.5	98.7 ± 8.6	176 ± 14.8 ^ab^	125 ± 11.5 ^bc^
TGs (mg/dL)	84.4 ± 7.9	73.4 ± 5.1 ^a^	188 ± 15.6 ^ab^	98.4 ± 8.6 ^bc^
CHOL (mg/dL)	74.5 ± 8.8	64.3 ± 5.7 ^a^	154 ± 14.6 ^ab^	88.3 ± 9.4 ^bc^
LDL-c (mg/dL)	39.8 ± 5.6	31.2 ± 4.1 ^a^	103 ± 13.2 ^ab^	63.4 ± 6.1 ^abc^
HDL-c (mg/dL)	18.7 ± 2.5	26.7 ± 5.4 ^a^	7.8 ± 1.4 ^ab^	16.5 ± 3.1 ^bc^
FFAs (µmol/L)	446 ± 39.5	378 ± 29.3 ^a^	943 ± 88.5 ^ab^	559± 3.1 ^abc^
Adiponectin (µg/mL)	38.6 ± 4.1	41.6 ± 4.7	19.5 ± 3.2 ^ab^	34.6. ± 5.3 ^abc^
Leptin (ng/mL)	13.4 ± 2.5	14.1 ± 2.3	32.2 ± 3.8 ^ab^	18.5 ± 4.1 ^bc^
TNF-α (pg/mL)	123 ± 11.7	119 ± 14.7	298 ± 31.2 ^ab^	178 ± 16.9 ^bc^
IL-6 (pg/mL)	15.6 ± 1.8	16.7 ± 2.8	48.3 ± 5.6 ^ab^	22.8 ± 3.6 ^bc^

Data are presented as means ± SD (*n* = 8/group). ^a^: vs. normal; ^b^: vs. morin-treated normal rats (50 mg/kg); ^c^: vs. HFD-fed rats.

## Data Availability

The datasets used and/or analyzed during the current study are available from the corresponding author upon reasonable request.
